# BicaudalD Actively Regulates Microtubule Motor Activity in Lipid Droplet Transport

**DOI:** 10.1371/journal.pone.0003763

**Published:** 2008-11-19

**Authors:** Kristoffer S. Larsen, Jing Xu, Silvia Cermelli, Zhanyong Shu, Steven P. Gross

**Affiliations:** Department of Developmental and Cell Biology, University of California Irvine, Irvine, California, United States of America; Institut Pasteur, France

## Abstract

**Background:**

A great deal of sub-cellular organelle positioning, and essentially all minus-ended organelle transport, depends on cytoplasmic dynein, but how dynein's function is regulated is not well understood. BicD is established to play a critical role in mediating dynein function—loss of BicD results in improperly localized nuclei, mRNA particles, and a dispersed Golgi apparatus—however exactly what BicD's role is remains unknown. Nonetheless, it is widely believed that BicD may act to tether dynein to cargos. Here we use a combination of biophysical and biochemical studies to investigate BicD's role in lipid droplet transport during *Drosophila* embryogenesis.

**Methodology/Principal Findings:**

Functional loss of BicD impairs the embryo's ability to control the net direction of droplet transport; the developmentally controlled reversal in transport is eliminated. We find that minimal BicD expression (near-BicD^null^) decreases the average run length of both plus and minus end directed microtubule (MT) based transport. A point mutation affecting the BicD N-terminus has very similar effects on transport during cellularization (phase II), but in phase III (gastrulation) motion actually appears better than in the wild-type.

**Conclusions/Significance:**

In contrast to a simple static tethering model of BicD function, or a role only in initial dynein recruitment to the cargo, our data uncovers a new dynamic role for BicD in actively regulating transport. Lipid droplets move bi-directionally, and our investigations demonstrate that BicD plays a critical—and temporally changing—role in balancing the relative contributions of plus-end and minus-end motors to control the net direction of transport. Our results suggest that while BicD might contribute to recruitment of dynein to the cargo it is not absolutely required for such dynein localization, and it clearly contributes to regulation, helping activation/inactivation of the motors.

## Introduction

Dynein is involved in many cellular processes, including mitosis, nuclear migration, mRNA transport, mitochondrial transport, golgi positioning, endosomal motion, transport of a variety of axonal and dendritic vesicles, IF motion, and the transport of pathogens such as herpes viruses. In many cases, it appears to play dual roles: first, it is essential for transporting particular organelles to specific locations, and second, once the organelles are appropriately localized, dynein plays a role in anchoring them there. Many of these cargos are transported along microtubules in a bi-directional fashion, involving the activity of a plus-end motor such as a kinesin family member and the activity of the minus-end motion dynein.

The regulation of this bi-directional transport, or even uni-directional dynein-based motion alone, is not well understood. However, past work has uncovered a role for the protein BicD in facilitating dynein function in a number of systems. In many of these systems, loss of BicD function results in mislocalization of the cargo. Most of these studies have not involved real-time imaging and analysis. Therefore, it has not been clear to what extent the loss of BicD function alters actual transport of the cargo in question, as opposed to what extent it plays an essential role in anchoring the cargo once it is appropriately delivered.

BicD was originally identified in *Drosophila*, in a mutant screen for dominant maternal-effect proteins [1]. BicD mutant embryos exhibited severe developmental defects including the loss of positional information defining the anterior region of the embryo and thereby giving rise to bicaudal embryos. It was later established that this phenotype resulted primarily from the aberrant localization of oskar mRNA, a posterior determinant which inhibits the anterior factors bicoid and hunchback [2]. Further studies showed that this improper localization resulted from altered dynein-based transport of the mRNA particles [3]. BicD has been demonstrated to be a component of dynein based transport via genetic analysis in Drosophila [2], [4], [5]. In addition to its role in mRNA transport, BicD has been established to play a role in nuclear positioning in *Drosophila* [6] and in golgi positioning in mammalian tissue culture [7], [8].

**Figure 1:**
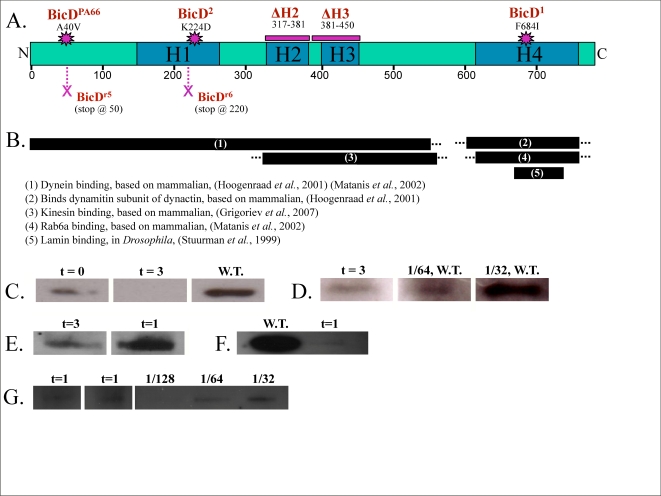
BicD structure and expression levels. A: Drosophila BicD is 782 amino acids in length. There are four heptad repeat motifs. The location and type of various BicD alleles used in this study are indicated. Starbursts represent point mutations, Xs represent premature stop codons, and horizontal bars represent specific domain deletions. B: A generalized map of the interacting regions of BicD. Dashed lines indicate an approximate position based on data from mammalian BicD homologs. Regions displayed represent published data and not necessarily minimal binding sites. C: Ovarian BicD levels in heat-shock rescued BicD^null^ ovaries are quite low compared to wild-type. Levels seen during repeat daily heat-shocks (t = 0), and three days post heat shock cessation (t = 3) are shown in comparison to wild type. Lanes were loaded with equivalent amounts of total lysate. D: Specifically examining ovarian BicD expression in t = 3 ovaries indicates expressions levels approximately 1% of that in wild-type. This is shown in comparison to a wild-type dilution series of lanes loaded with 1/32_nd_ and 1/64_th_ the amount of total protein loaded for the t = 3 lane. E: A comparison of embryonic BicD expression between heat shock rescued embryos whose mothers have been off heat shock for one versus three days. F: The decrease in ovarian levels results in a decrease in embryonic levels: a comparison of BicD levels in embryos from OrR females (left) and BicD^null^/t = 1 females (right). Lanes were loaded with equivalent amounts of total embryo lysate. Signal was detected with mixed anti-BicD (4C2, 1B11). G: Quantitation of overall reduction of BicD in t = 1 embryos relative to the wildtype. Both t = 1 lanes were loaded with equivalent amounts of total embryo lysate; OrR lanes for comparison were loaded with 1/32, 1/64, or 1/128th the relative amount of total sample loaded in the t = 1 lanes. The t = 1 lanes have approximately the same signal strength as the OrR lysate diluted 1/64, suggesting that in the t = 1 embryos there is approximately 1.6% of wildtype BicD levels. Signal is detected with mixed anti-BicD antibodies (4c2, 1B11).

Molecular studies have provided some insight into BicD's function. In mammals, two homologues of Bicaudal D, BICD1 and BICD2, are present [8], [9]. BicD is highly conserved, albeit there is only one isoform in *Drosophila*. BicD is believed to exist *in vivo* as a homodimer [10], [11]. Its C-terminus appears to bind to cargos [12], while also being known to foster specific dynein/dynactin interactions [8], [13] (Fig 1B). BicD is generally classified as having four well-defined coiled-coil regions (Fig. 1A) comprised of multiple heptad repeats–without any other apparent motifs [11]. Overall, BicD has a flexible comma shaped structure with one large and one small globular domain and an apparently flexible linkage in between [10]. The large globular domain is believed to be at the C-terminus where binding to the cargo occurs.

Mammalian BicD can directly interact with both Dynein and Dynactin, and because a cargo linked N-terminal fragment of BicD can tether dynein and subsequently induce dynein-based transport [12], it is believed that BicD may link dynein to the cargo. This is consistent with the studies of BicD's role in *Drosophila* mRNA transport, where overexpression of BicD leads to increased association of dynein with the mRNA particles [14]. Thus, one currently favored hypothesis is that BicD is a structural protein required for dynein localization. Tools have not existed to investigate the possibility that it might play important regulatory roles as well.

Here, we investigate BicD's function within the context of bi-directional lipid droplet based transport. In contrast to other systems where BicD has been studied, the particular strength of the droplets is that it is possible to image them with high temporal and spatial resolution, allowing a careful study of how loss of BicD affects the properties of motion. Further, the possibility of biochemical purifications of droplets means that it is possible to investigate additional aspects of BicD's function. Importantly, while the droplets always move bi-directionally, they undergo developmentally controlled changes in net transport[15]. Early in development (nuclear division cycles 11–13) the droplets move but with no net transport (“phase I”). Then, in early cycle 14, the droplets move basally (net plus-end transport, “phase II”). Finally, in later cycle 14 there is net minus-end transport (“phase III”). Thus, by studying changes in droplet-bound proteins at different times during development, it is in principle possible to gain insight into how transport is regulated[16].

We show that while BicD may help to recruit dynein to the cargo, it is not absolutely essential for at least partial dynein recruitment. Also, its role is more complicated than performing this task alone: during development, the majority of BicD leaves the droplet, but the dynein stays, and this subsequent departure of BicD does not obviously alter dynein function. Our measurements uncover a new role for BicD, in directly or indirectly contributing to plus-end motor activity. Finally, analysis of an intriguing BicD point mutation uncovers a clear ongoing temporally changing regulatory role for BicD.

## Results

**Figure 2:**
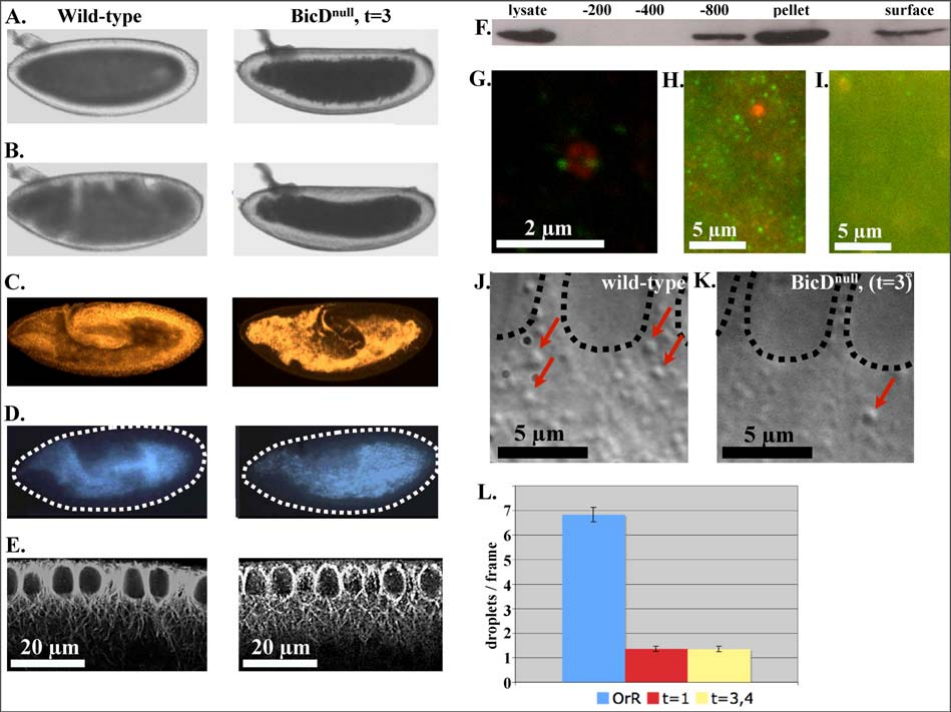
Lowering BicD expression has a net effect on lipid droplet transport. A and B: Transmitted light images of developing BicD^null^ (t = 3) embryos indicate proper lipid droplet clearing in phase II (A), but an inability to recloud in phase III (B). C: Prior to Phase 3, during germband extension, the neutral-lipid specific dye Nile Red shows a net position of lipid droplets which is very different between wild-type and BicD^null^ embryos. D: During germband extension, DAPI autoflouresence of yolk vesicles does not indicate a significantly different positioning of these cargoes. Dashed lines delineate the embryos edge. E: No gross defects are observed in the MT structure of mutant embryos, as exemplified by the above image of MT immunoflouresence. Both images show microtubules passing from above, between, and below the syncytial nuclei. F: BicD localizes to the layer of lipid droplets which spin up through a sucrose gradient during centrifugation from an initial embryo lysate due to their bouyent density. Equal volumes of extracted sample were loaded per lane of the western. BicD signal can be found in the pellet, the layer immediately above the pellet (-800), and at the surface where lipid droplets coalesce. G: Confocal analysis of whole fixed embryos shows some BicD signal (green) close to droplets (red). Red signal represents the droplets specific marker LSD2. Lipid droplets generally average approximately 500 nm in size. H and I: With less magnification, standard fluorescence microscopy shows significant punctual staining of the cytoplasm in Phase I wild-type embryos (H) but not in Phase I, BicD^null^ (t = 3) embryos (I). Here, green is BicD, and red is the lipid droplet specific protein LSD2. J and K: Phase II DIC images show a clear difference in the number of lipid droplets near the nuclei. Dashed lines specify the position of the nuclei and red arrows are shown indicating the position of several lipid droplets. L: Automated analysis of the number of droplets observed per frame, in phase II, confirms the apparent decrease in droplets seen at the periphery of the embryo in ‘t = 1’ and ‘t = 3–4’ mutant backgrounds.

Because BicD is reported to play a role in either the transport or positioning of multiple organelles, and we have already established that our droplets are moved by dynein [17], we were interested in determining if BicD also played a role in lipid droplet transport. Globally, the distribution of lipid droplets in the embryo can be visualized using the neutral-lipid specific dye, Nile Red (Fig. 2C), and also by transmitted light microscopy (Fig. 2A, B).

To investigate whether BicD played a role in droplet motion, we first wanted to decrease the overall amount of embryonic BicD, and determine whether this had any effect on droplet transport. Because the proteins in early embryos are predominantly maternally derived, and the complete loss of BicD is lethal, it has not been possible to generate embryos entirely lacking in BicD. Instead, we used a strategy based on a previously developed heat shock rescue technique to deliver just enough wild-type BicD to rescue otherwise BicD^null^ females [5]. Upon cessation of heat-shocking, ovarian BicD levels fell quickly. Continued for one hour per day, heat shocks of otherwise null mothers produced significantly less ovarian BicD than did wild-type mothers (Fig. 1C). Also, by three to four days post heat shock (t = 3,4), ovarian BicD levels appeared to be slightly less than 1/64^th^ wild type levels–i.e., approximately 1% of wild type (Fig. 1D). This loss of ovarian BicD resulted in a decrease in embryonic BicD (Fig. 1F); BicD in t = 1 embryos was approximately 1.6% that in wildtype embros (Fig. 1G). Additionally, in the low BicD- embryos, BicD levels continued to decrease as a function of time from heat shock: there is an approximate 6-fold difference between BicD levels at one day post heat shock (t = 1) and three to four days post heat shock (t = 3,4) (Fig. 1E). Thus, in t = 3,4 embryos, we calculate that BicD levels are ∼0.267% of wild-type, i.e. have been reduced more than 300×.

Using this technique therefore allowed us to achieve very low–and tunable–embryonic BicD levels. Examining the droplets in these embryos, we discovered that the loss of BicD had a number of effects. First, even when droplets are developmentally ‘clearing’ from the embryo's edge (in Phase II), there were many fewer droplets obvious in this peripheral region of the low BicD embryos (Fig. 2K, L) as compared to wild-type (Fig. 2J, L). This could simply reflect a redistribution of droplets to the embryo center (where we are unable to quantify droplet distribution), or could reflects an overall decrease of embryonic droplets present in the embryo in this background. Second, loss of BicD severely altered the global droplet distribution (Fig. 2A–C): droplets cleared in phase II, but failed to re-cloud in phase III.

To further understand how this altered global distribution of droplets came about, we considered a number of possibilities. The first was that loss of BicD led to an altered microtubule network in the embryos. To test this idea, we used confocal microscopy to visualize the microtubule network in fixed intact embryos. While the microtubules in the mutants appear slightly more disorganized, variation between different embryos is seen in the microtubule organization in both wild-type and mutants, and we observed no gross defects in the microtubules (Fig. 2E). To further test the viability of the microtubule network, we examined the distribution of another cargo, yolk vesicles, whose distribution was previously shown[18] to depend on intact microtubules. Their distribution was approximately normal (Fig. 2D). Thus, we hypothesize that the altered lipid droplet distribution was due to either altered droplet transport, or impaired ability to anchor the droplets once they were delivered.

### BicD likely contributes to appropriate droplet transport directly, through a role on the droplets

Past studies have shown that the N-terminal portion of mammalian BicD can co-precipitate components of both the dynein and dynactin complexes [8], [12]. Also, BicD homologs have been shown to directly bind dynein intermediate chain [13], the dynamitin subunit of dynactin [8], and kinesin [19] (see also Fig. 1B). This suggests that, for lipid droplets as well, BicD might facilitate appropriate transport via direct interaction with the transport machinery. To test whether BicD might play a direct role in droplet transport, we first investigated whether BicD was indeed present on the droplets. A first analysis involved purifying droplets, by spinning lipids from an embryo lysate through a simple sucrose step gradient. We saw, via western, that BicD signal was found on the lipids spun to the surface and at or near the pellet. However, it was not detectable in the fractions immediately below the surface lipids (Fig. 2F). This suggested that BicD was not simply everywhere in the gradient. Using the same antibodies for immunoflouresence (in the whole embryo) clearly demonstrated a general lack of BicD signal in the t = 3 background as compared to wild-type (Fig. 2H, I). In wild-type embryos strong punctae were observed, which were predominantly lacking in the BicD-low mutant. Some of these punctae were found close to lipid droplets (Fig. 2G). Subsequent larger volume and higher speed lipid purifications (used our previously developed protocol to purify lipid droplets) confirmed BicD's presence in the droplet fraction. Extensive characterization of the purification protocol [20] has established that the lipid droplet fraction does not have large contaminants. The presence of BicD on the droplets is consistent with a direct role in facilitating/regulating transport.

### Lowered embryonic BicD levels alter individual droplet motion

**Figure 3:**
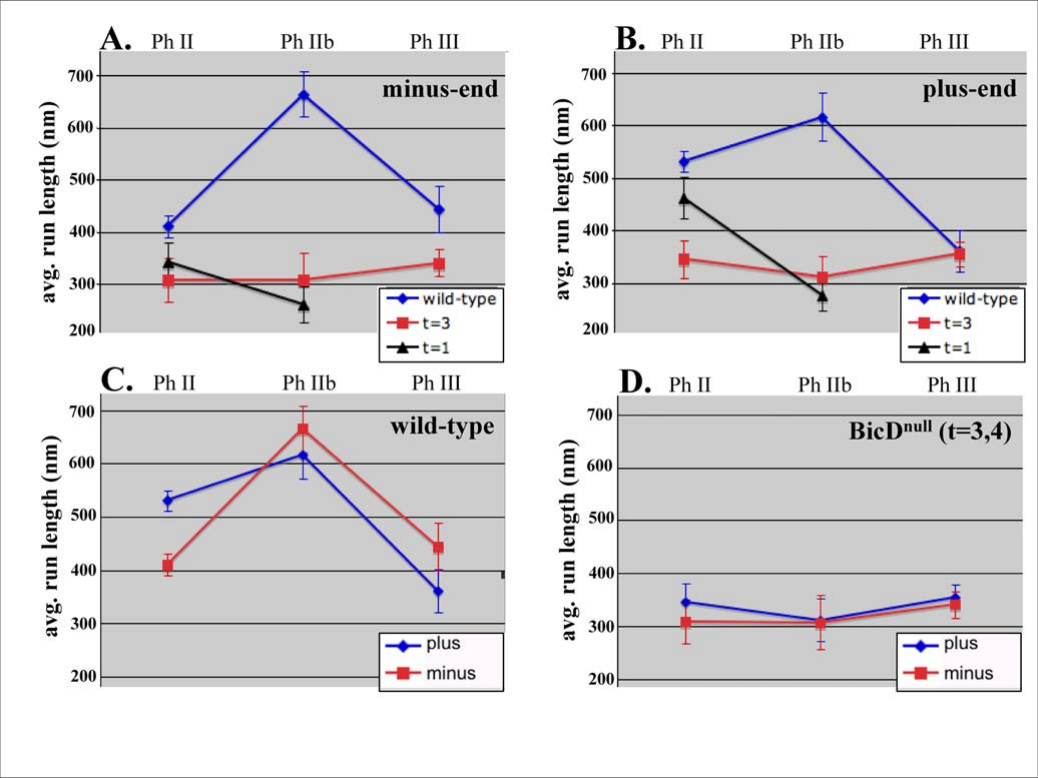
Droplet distribution is altered due to BicD-induced changes in Droplet motion. A: BicD contributes to correct minus-end motion. In the t = 3,4 null background, minus-end directed run lengths are shorter in all three phases under consideration. While a clear run length peak is present in phase IIb of wild-type, there is no such peak in the null. B: Comparison of plus-end directed run lengths shows that BicD affects this direction of transport as well, although, not in phase III (B). Note the difference between t = 1 and t = 3 plus-end run lengths (t-test, p = 0.049) in phase II (A). A similar difference is not apparent in the minus-end direction (t-test, p = 0.56) of transport (B). C and D: The net reversal of directionality seen to occur in phase IIb for wild-type (C) does not occur in the mutant (D).

To differentiate between an actual transport problem and simply a problem tethering droplets once they are delivered, we analyzed the motion of droplets with high temporal and spatial resolution. While a global analysis suggested a role for BicD in controlling the Phase III distribution of lipid droplets, altered transport in Phase II could in principle contribute to phenotypes observed in Phase III. We therefore recorded and analyzed droplet motion in both phase II and III. We initially examined motion in Or-R embryos (wild-type, phase II, N = 9 embryos, n = 305 droplets tracked; phase IIB, N = 3 embryos, n = 143 droplets; phase III, N = 2 embryos, n = 45 droplets), t = 1 embryos (low BicD, phase II, N = 11 embryos, n = 52 droplets; phase IIB,N = 9 embryos, n = 18 droplets) and t = 3,4 embryos (BicD∼null, phase II, N = 7 embryos, n = 19 droplets; phase IIB, N = 5 embryos, n = 12 droplets; phase III,N = 5 embryos, n = 50 droplets). Comparing wild-type motion to motion in the t = 3 background, we find that loss of BicD shortened both plus-end and minus-end run in phase II, but only minus-end runs in phase III (Fig. 3A, B). While we presume that decreased overall embryonic BicD levels result in decreased droplet-bound BicD levels (consistent with the disappearance of BicD puncta in the low-BicD embryos, Fig 2), we cannot directly show this because we cannot purify droplets from the limited number of developing mutant embryos.

We also discovered a new intermediate phase of transport, phase IIb, wherein there is a large up-regulation of both plus- and minus-end directed run lengths in wild-type (Fig. 3C). Chronologically, this phase of transport lasts for ∼10 minutes, and appears to coincide with the reversal of the net direction of transport. In the t = 3 (∼null) embryos, this phase does not occur: the quantitative parameters of droplet motion in phase II t = 3 embryos are the same as for phase IIb t = 3 embryos (t-test, p = 0.56). Thus, the presence of BicD is somehow required for the regulatory changes involved in control of this set of changes in droplet motion. Intriguingly, in the t = 1 embryos where there is a small yet significant residual amount of BicD (Fig. 1E), some form of the Phase II→IIb transition occurs, but its form is different: in the t = 1 embryos, the plus-end run lengths go down instead of up (Fig. 3). This is statistically obvious for plus-end runs (t-test, p = 5.3×10^−4^; Fig. 3B), and there is a hint that this might be true for minus-end motion as well (Fig. 3A), although we cannot be certain because the effect in the minus-end direction is small, and within uncertainty (t-test, p = 0.12). Regardless, the decrease in plus-end run lengths during the phase II to IIb transition of the t = 1 embryos (t-test, p<0.04), is in the opposite direction from the change seen in wild-type where run-lengths increase (t-test, p<0.04), suggesting that the exact levels of BicD present may play an important role during this transition under normal circumstances in the wild-type. Supporting the hypothesis that plus-end transport may be quite sensitive to BicD levels, we note that in phase II, minus-end motion in the t = 1 and t = 3 backgrounds is approximately the same (t-test, p = 0.56; Fig. 3A), but the small amount of additional BicD present in the t = 1 embryos is enough to significantly improve Phase II plus-end motion above that found in the t = 3 embryos (t-test, p = 0.049; Fig. 3B).

### Droplet-bound BicD levels are temporally changing, and regulated

**Figure 4:**
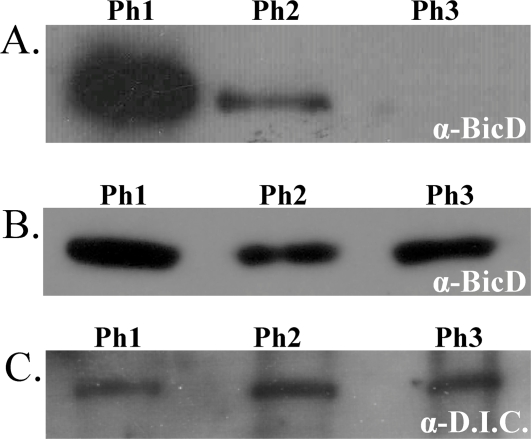
Levels of BicD on the lipid droplets decrease sharply with time. A: Lipids purified from staged one hour collections were probed for BicD and showed a large decrease in the amount of BicD on the droplet from phase I to III. Each lane was loaded with an equivalent amount of purified protein. B: During this same time, embryonic BicD levels are approximately constant. Each lane was loaded with an equivalent amount of total protein. C: Phase specific lipid purifications suggest that dynein levels on the droplets are approximately constant throughout phase I–III. Equivalent amounts of protein were loaded per lane.

Since the t = 1/t = 3 experiments suggested that BicD levels may be important, we investigated whether droplet-bound BicD levels changed in conjunction with normal developmental changes in droplet motion. To do this, rather than purifying droplets from embryos in a 4-hour collection, we used a previously developed approach [16], [20] to do temporally staged purifications, enabling us to investigate whether there were any changes in droplet-bound BicD levels as a function of time. To our surprise, levels of BicD on the droplet decreased precipitously from phase 1 to 3 (Fig. 4A). This result was repeated multiple times and sample volumes per lane were normalized for total protein each time.

Droplet-bound BicD levels could be actively controlled, or could passively reflect changing embryonic levels of BicD. To differentiate between these possibilities, we did temporally controlled collections, as described above with respect to droplet purifications. However, rather than purifying the droplets, we simply looked at overall embryonic levels of BicD. We found (Fig. 4B) that embryonic BicD levels are relatively stable during this period, suggesting that the amount of droplet-bound BicD is somehow directly regulated.

Given the above t = 1/t = 3 results, and the observed changes in BicD levels, it seems likely that regulated changes in droplet-bound BicD levels could functionally contribute to the regulation of droplet transport and net directionality. Past studies reported that, when purposely attached to a cargo, an N-terminal fragment of mammalian BicD alone could recruit dynein to a given cargo [12]. Therefore, one appealing scenario is that droplet-bound BicD tethers dynein to the cargo, and thus droplet-bound BicD levels directly control transport by determining the amount of cargo-bound dynein. Then, regulating droplet-bound BicD levels could be used to regulate droplet-bound dynein levels, and potentially dynein activity. To investigate this possibility, we examined the amount of dynein bound to the droplets using staged lipid droplet purifications. DIC is hard to detect in western blots, so we have significantly more variability than for many other proteins. Nonetheless, we do not generally detect an obvious change in droplet-bound dynein levels as a function of developmental stage (Fig. 4C), suggesting there are likely not dramatic changes in the amount of dynein bound to the droplets in different developmental phases (though these measurements are not sensitive enough to detect subtle changes). Additionally, wild-type minus-end run lengths are approximately constant between phase I, II, and III [15], although they do increase briefly in the newly discovered phase IIb (Fig. 3C). Also, minus-end stalling forces (which reflect the number of engaged motors) have been shown to be higher in phase II than in phase I [15], although droplet-bound BicD is much lower in phase II than in phase I. Since the drastic fall in droplet BicD levels does not clearly coincide with altered minus-end run lengths in wild-type, or in reduced minus-end stalling forces, it seems unlikely that in phases I through III the majority of BicD would be playing an important role in tethering dynein to the cargos.

To test the idea that BicD may not be absolutely essential for minus-end motility, we compared droplet motion in low (t = 1) and ∼null (t = 3) BicD expression backgrounds. We observed minus-end motion that was statistically similar in the low and extremely low BicD backgrounds, despite the approximate six-fold difference in BicD expression (Fig. 1E). This suggests that by the time we reach t = 3 BicD levels, we are observing minus-end directed transport on droplets that are functionally null for BicD, consistent with the idea that BicD may not be absolutely required for droplet motion. However, if BicD is important for initial recruitment of dynein, we might expect that the percentage of droplets moving to be decreased. Our automated analysis found that, in phase II, the number of moving droplets per frame was essentially the same between the t = 1 and t = 3 backgrounds, but quite reduced relative to the wild-type case (see below). These observations are consistent with a model that there is a basal amount of dynein that makes it to the droplets in a BicD-independent fashion, but that some of the droplet dynein initially is recruited via BicD (during embryogenesis), and that only droplets with dynein are able to transport themselves into the forming embryo. However, other interpretations are also possible (see discussion).

### Mutant analysis to gain insight into BicD's functions

**Figure 5:**
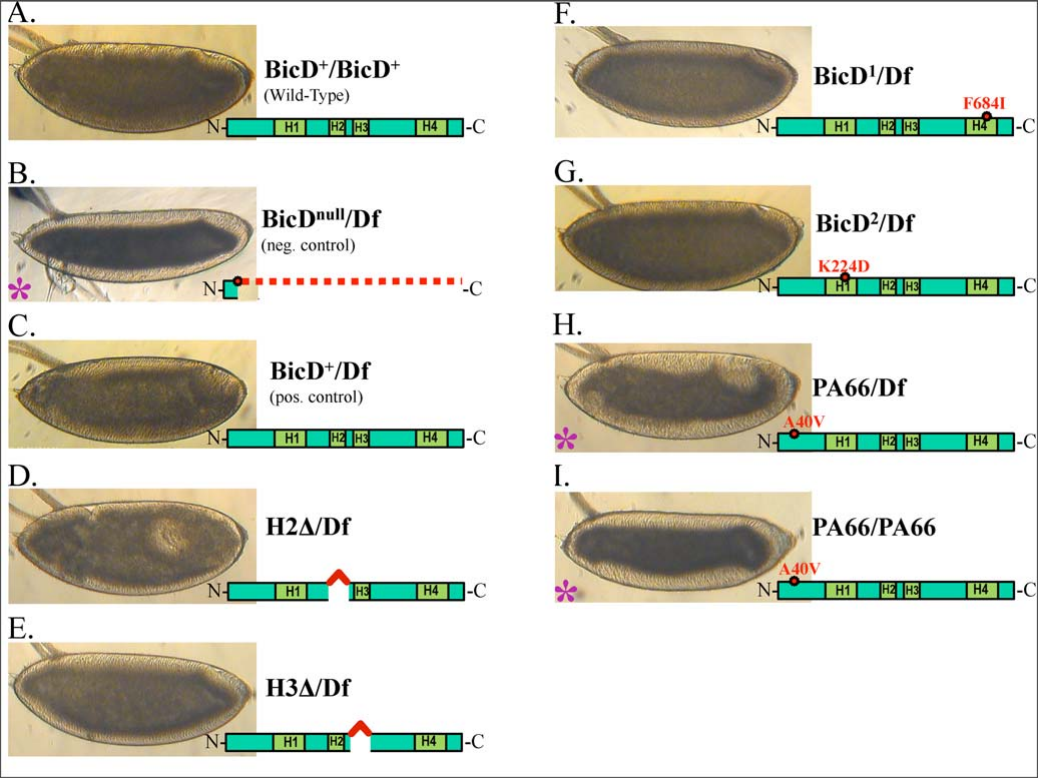
Examining BicD alleles for the ability to rescue the reclouding defect of a low BicD background. A–I: Alleles comprised of domain deletions, point mutations, and premature stop codons were examined. B–C: Negative and positive controls are shown for comparison. All alleles appeared to rescue (C–G), with the exception of the point mutation BicD^PA66^ (H–I). Rescuing with two copies of BicD^PA66^ displayed a continued failure to rescue (I). E: On average, there is the suggestion that there may be only a partial rescue phenotype seen in the BicD^H3Δ^ rescue background. Images with purple asterisks are those with a BicD-null phenotype.

Given the sudden BicD-dependent change in motion from phase II to IIB, it is possible that BicD has two roles—an initial structural one, possibly recruiting dynein as previously discussed by others, and a latter dynamic regulatory role, once the dynein is recruited. To further investigate this idea, and identify possibly separable functions of BicD, we undertook a structure-function study of a number of BicD mutants. We used a genetic strategy, wherein we evaluated whether specific mutations in BicD affected functions important for lipid droplet motion. Experimentally, our goal was to determine which alleles were capable of rescuing an expected reclouding defect (Fig. 5B) in embryos from female flies expressing very low levels of BicD. The rescue background, i.e. P{HA::BicD}/+; BicD−/BicD−, was expected to express approximately 6% wild-type levels of BicD based on previous studies [11]. Our strategy involved making female flies, which in addition to the low levels of BicD, had BicD alleles that could potentially “rescue” the global reclouding defect.

Using the female flies heterozygous for a HA-tagged BicD transgene (a gift from the lab of Dr. Ruth Steward), we examined several alleles comprised of small deletions and independent point mutations for their ability to rescue. Point mutants BicD^1^ (F684I) and BicD^2^ (E224K ) appeared to strongly rescue reclouding (Fig. 5F, G). A deletion of the second heptad repeat, from the N-terminus, rescued as well (Fig. 5D). A deletion of the third heptad repeat rescued, albeit perhaps not fully (Fig. 5E). By far, the clearest failure to rescue was provided by the A40V point mutant BicD^PA66^ (Fig. 5H, I). Thus, we decided to focus on a deeper understanding of the BicD^PA66^ allele. As part of this analysis, we tracked and analyzed droplets moving in BicD^PA66^ embryos in both phase II (N = 9 embryos, n = 122 droplets tracked), phase IIB (N = 5 embryos, n = 183 droplets tracked), and in phase III (N = 8 embryos, n = 236 droplets tracked).

### Further studies on the BicD^PA66^ allele

**Figure 6:**
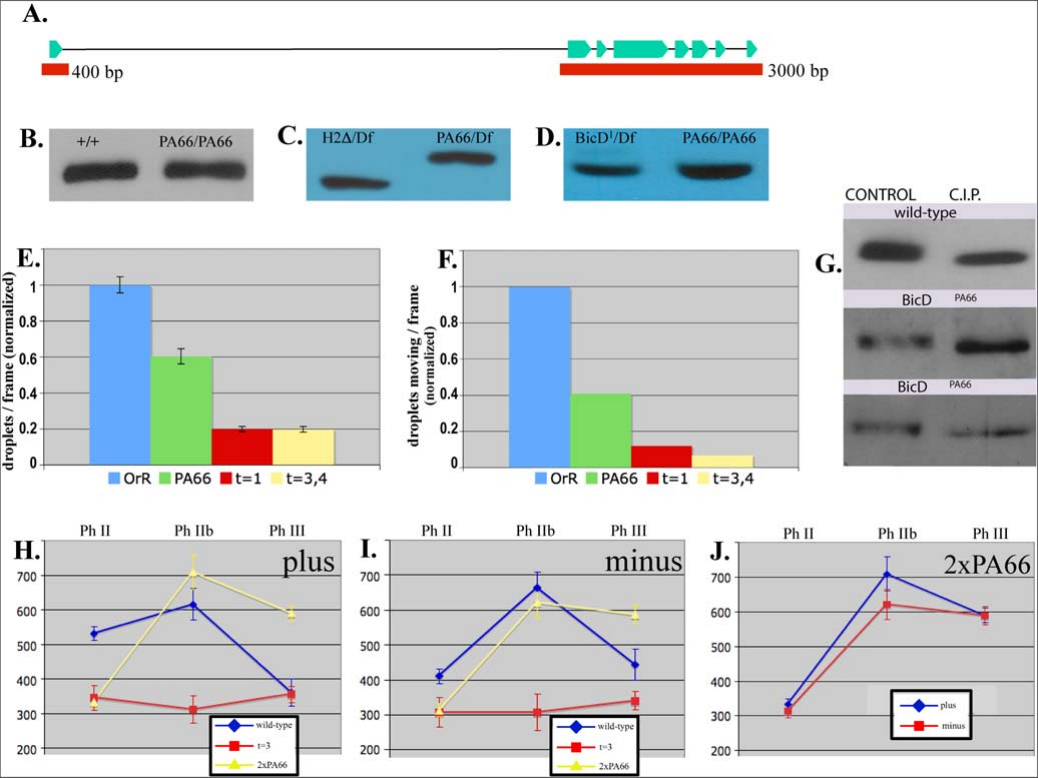
An analysis of the BicD^PA66^ allele's failure to rescue. A: Sequence analysis of the BicD^PA66^ allele confirmed, as expected, that there was a single point mutation resulting in a single divergence (A40V) from the wild-type amino acid sequence. Light blue regions represent exons. Introns are represented by the intervening thin black lines. Red bars represent the two fragments upon which sequence analysis was carried out. B–D: BicD^PA66^ expression levels are similar to wild-type (B). BicD^PA66^ also showed similar expression to other alleles that were capable of rescue such as BicD^1^ and BicD^H2Δ^ (C, D). Equivalent amounts of total embryo lysate were loaded per lane for each independent comparison (B–D). E: In phase II, BicD^PA66^ analysis identified a total number of droplets per frame intermediate between the wild-type and the null (‘t = 1’, ‘t = 3,4’) values. F: Also in phase II, the identified number of moving droplets in the BicD^PA66^ background was intermediate to the lower null values and the higher wild-type values. G: In the embryo, both BicD^+^ and BicD^PA66^ appear to be phosphorylated as indicated by the resulting shift following phosphatase (C.I.P.) treatment. H–J: Particle tracking of BicD^PA66^ showed that phase II run lengths were identical to the BicD^null^ (t = 3) for both directions of transport (t-test, p>0.75 for both directions) while phase IIb run lengths were similar to wild-type (t-test, p>0.17) for both directions of transport (H, I). Phase III run lengths did not come back down from the phase IIb peak as seen in wild type. BicD^PA66^ displayed an inability to reverse the net direction of transport, while also displaying a slight bias for net plus-end directed transport (J).

Since BicD levels are important for overall function, one possibility was that the point mutation altered protein expression or stability. To test this possibility, we first used western blot analysis to determine BicD levels in the PA66 mutant embryos. We found that BicD^PA66^ expression levels were similar to wild-type (Fig. 6B). Further, rescuing with two copies of BicD^PA66^ instead of one displayed a continued reclouding defect (Fig. 5I). BicD^PA66^ also displays expression similar to the H2Δ and BicD^1^ alleles (Figs. 6C, D), both of which clearly rescue (Fig. 5D, F). Combined, these observations suggest that lowered BicD levels could not account for the failure of the BicD^PA66^ allele to rescue the altered droplet distribution.

A second possibility is that BicD function is important for appropriate arrangement of the microtubule cytoskeleton, so that the altered droplet transport in the BicD^PA66^ embryos could reflect a generalized microtubule defect. However, this is unlikely here because BicD^PA66^ and BicD^null^ (t = 3) embryos have nearly identical tracking phenotypes in phase II (t-test, p>0.75 for both directions; Fig. 6H, I), and confocal images of phase II microtubules in BicD^null^ (t = 3) embryos generally appeared fine (Fig. 2E). Additionally, the short run lengths in BicD^PA66^ phase II embryos transition to long wild-type run lengths of phase IIb within ∼4 minutes, which would imply a rapidly repaired microtubule array, which seems unlikely. Overall, it seems unlikely that the short run lengths in phase II of BicD^PA66^ and the t = 3 null are attributable to microtubule defects.

A third possibility is that the failure of the BicD^PA66^ allele to rescue motion reflects the impaired localization of BicD^PA66^ to the droplet, due to the PA66 point mutation. To investigate this possibility, we considered a number of lines of evidence. First, others' structural studies have suggested that BicD typically attaches to cargos via its C-terminal domain, but our sequencing analysis (Fig. 6A) indicated that the location of the point mutation is indeed as expected, in BicD's very N-terminal domain, far from the C-terminal cargo-attachment region. Further, the analysis found no additional mutations (e.g.in the C-terminal portion of the protein) that could account for altered localization. Second, early in development (before phase II, when we analyze motion), BicD may recruit some portion of the Dynein to the droplets. This hypothesis is consistent with past reports, and also with the observation that in phases II and IIb, the number of moving droplets in BicD^null^ (t = 3, 4) was decreased by approximately 10× as compared to wild-type (Fig. 6F). With this in mind, we looked at the number of droplets moving in the PA66 background, and while it was less than in wild-type, it was roughly 3× to 4× more than in the null (t = 3, 4) background (Fig. 6F). Since the presence of BicD with the PA66 mutation resulted in significantly more moving droplets than for the BicD^null^, it suggests that the PA66 form of the protein still has a reasonable ability to recruit dynein to the droplets, consistent with the hypothesis that BicD^PA66^ is indeed able to localize to the droplets (at least early in development). Unfortunately, because they are quite few in number, we cannot use biochemistry (e.g. droplet purifications) on the BicD^PA66^ embryos to confirm that the BicD^PA66^ protein localizes to the droplets. Further, immunolocalization studies were inconclusive because BicD serves many roles, so we observe punctae both close-to and away from the droplets in both wild-type and BicD^PA66^ mutant embryos (not shown). However, from a functional point of view, tracking analysis indicates that droplet motion (and hence BicD^PA66^ function) in Phase IIb is similar to wild type (Fig. 6H, I). This suggests that, at least in phase IIb, BicD is still physically present on the droplet. Similarly, motion continues to be good in phase III BicD^PA66^ embryos. In phase III, BicD^PA66^ run lengths of both plus and minus direction are noticeably *longer* than wild type (Fig. 6H, I). Combined, these observations suggest that BicD's ability to possibly initially recruit dynein to the cargo is separate from its latter ability to regulate or alter transport. Rather than suggesting that the A40V mutation of BicD^PA66^ simply alters its localization, the data is consistent with the hypothesis that this mutation leaves BicD's initial ability to recruit Dynein partially intact, but alters BicD's function by somehow changing its ability to later regulate motion. Such a hypothesis would be consistent with the point mutation being localized in a portion of the protein previously reported to interact with dynein [12].

## Discussion

Past studies have found numerous roles for BicD in contributing to distinct dynein-mediated processes, but the exact mechanism through which BicD contributes to appropriate minus-end transport is not yet well established. However, evidence in a number of systems suggests that BicD may be able to play a role in recruiting dynein to the cargos. These past studies are important, but rely heavily on over-expression studies, and/or expression of truncated forms of the protein, and so leave open the question of whether BicD levels or activity are changed as part of the usual regulatory process. In addition, even accepting that BicD is important as a dynein recruiter, there has been open the question of whether BicD plays any continuing regulatory roles, distinct from making sure that the transport complex is appropriately set up. Our studies have investigated these questions within the context of bi-directional lipid droplet motion in *Drosophila* embryos.

### BicD appears not to function only as a static dynein tether

Our data is consistent with the hypothesis that BicD plays a role in recruiting dynein to the cargo: loss of BicD function leads to many fewer droplets present in the embryo, and hence fewer moving droplets, consistent with a role for BicD in recruiting dynein to droplets during embryogenesis, allowing them to be subsequently delivered into the egg. However, some dynein recruitment is BicD-independent, because the same number of droplets make it into the embryo in the t = 1 and t = 3 embryos (and approximately the same number of droplets are moving in these two backgrounds) as determined from tracking analysis, in spite of the 6× difference in BicD levels, and the fact that in the t = 3 embryos BicD levels have been reduced ∼375× relative to the wildtype, so it seems unlikely that there is still enough BicD remaining to be functional. This finding is consistent with past work investigating BicD's role in mRNA transport, which also concluded that BicD was not absolutely essential to recruit dynein [14]. Although our favored interpretation is that BicD plays a role in dynein recruitment, past work [21], [22] suggested that in ovaries of BicD-mutant mothers, the microtubule cytoskeleton can be impaired, resulting in improper transport of components into the developing embryos. Such disorganization could contribute as well to the apparent decrease in droplets present in the BicD-mutant embryos. Overall, although there are fewer droplets apparently present in the mutant embryos, the percentage of droplets present that actually move is approximately the same for all the genotypes, at ∼10%. We also note that there remains the possibility that some of the apparent decrease in embryonic droplets reflects increased basal droplet localization, to a central area of the embryo where droplet quantitation is difficult.

Nonetheless, numerous lines of evidence suggest that either a rather small portion of the available BicD tethers the dynein, or that whatever role BicD plays in dynein recruitment occurs in a temporally limited fashion. Loss of almost all the droplet-bound BicD (Fig. 4A) due to normal developmental regulation does not result in any loss of dynein from the droplets, as judged either by western blot, or stalling forces (we've shown previously[23], [24] that stalling force is roughly proportional to the number of engaged motors), or mean minus-end travel distances. A limited duration for BicD to help assemble the motor complexes is consistent with previous mRNA studies that suggest that the nature of the complexes moving the mRNA particles is sensitive to BicD levels when the complexes are formed, but not afterward [14].

### BicD contributes to dynamic regulation of droplet motion

We find that approximately the same number of droplets are moving in both of the very low-BicD backgrounds (Fig. 6F), although western blotting of embryos shows that there is ∼6× more BicD in the t = 1 background relative to the t = 3 background. This suggests that at such low levels of BicD, BicD can no longer play a significant role in recruiting dynein to the droplets (if it could, one would expect many more moving droplets in the t = 1 embryos). Thus, we hypothesize that there is a BicD-independent pathway for some dynein to get on to the droplets. Assuming that this hypothesis is true, i.e. that the levels of BicD present in both the t = 1 and t = 3 embryos are indeed too low to facilitate dynein recruitment, the observation that motion is altered between the two suggests that BicD likely has an additional regulatory role, independent of simple dynein recruitment, and that this regulatory role is affected by the amount of BicD present on the cargo. The existence of this dynamic regulatory role is further confirmed by the interesting phenotype of motion in the PA66 background, where the mutant form of the protein allows a dramatic change in droplet motion between phase II and IIb, but the subsequent down-regulation of transport expected between phase IIB and III fails to occur. A model for how the point mutation affects BicD's regulatory role is described below.

### BicD plays a role in kinesin-mediated travel

In addition to the evidence presented above supporting roles for regulation of minus-end transport, our data shows that loss of BicD function leads to altered plus-end motion, suggesting a new role for BicD: regulating plus-end transport. Our past studies suggest a strong coupling between plus-end and minus-end motion, so one question is whether the observed effects are indirect, and reflect a primary role in minus-end transport, with the altered minus-end transport resulting in plus-end transport. While we cannot be certain, we believe it is more likely that BicD plays a role in direct regulation of plus-end transport. First, recent work found that the middle portion of BicD interacts with kinesin via 2-hybrid [19]. When published, the functional significance of the interaction was unknown, but our data supports the hypothesis that it may be important. Our recent work[24]establishes that plus-end droplet motion is driven by kinesin-1, so it is unlikely that the effects of BicD on plus-end motion are simply due to its effects on dynein, although recent work suggests that this may be the case for some mRNA transport[25]. Second, the large phase IIb run length peak is absent in the t = 3 null, consistent with the hypothesis that BicD can acutely up-regulate both directions of transport. Third, we noted above that plus-end transport appears to have higher dosage sensitivity than minus-end transport to BicD levels (Fig. 3A, B).

Regardless of the exact mechanism, our studies show that BicD plays an important role in regulating the relative contribution of plus-end versus minus-end motion. Although run lengths are very long in phase III of the PA66 background, a reversal of the net direction of transport is not observed (Fig. 6J). Thus, the mutant analysis indicates that BicD's role in balancing plus-end versus minus-end motion is functionally separable from its role in facilitating overall upregulation of transport, with the resulting long runs in each direction in Phase IIB (Fig. 6H, I).

### A model for BicD function, incorporating findings from the PA66 point mutation

BicD^PA66^ is interesting in that the altered protein maintains the ability to up-regulate the high phase IIb run lengths, yet cannot regulate net directionality. If phase IIb run length up-regulation requires BicD, why are run lengths in phase II of the BicD^PA66^ background similar to the t = 3 null? In principle, BicD could be absent from the droplets in phase II and then rapidly accumulate at the onset of phase IIb, but this seems unlikely given that such a recruitment of BicD to the droplets in Phase IIB would go counter to the continued drop in droplet-bound BicD levels which usually occur during development.

**Figure 7:**
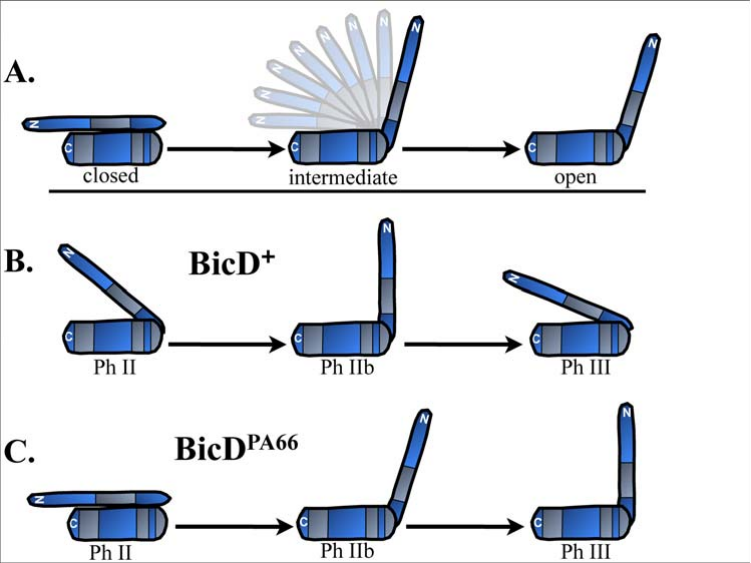
A proposed model for the proper regulation of BicD and the effect of the PA66 lesion. A: Past studies have indicated that BicD has a flexible linker region between its two globular domains (Stuurman *et al.*, 1999) and that an intramolecular interaction of the N- and C-terminal domains might represent a closed/inactive state (A, left) that can open to interact (A, right) with other regulatory components (Hoogenraad *et al.*, 2001). B: Wild-type BicD may regulate its active state by either controlling how frequently it alternates between a completely open/active vs. a completely closed/inactive state or through an ability to take an intermediate conformation. Therefore, intermediate conformations shown for wild-type (B left, B right) are simply intended to suggest that BicD molecules are not locked in one state or the other. Yet, the open stature of BicD shown for phase IIb of wild-type (B, middle) is intended to suggest a relatively open/active state. C: The A40V lesion of BicD^PA66^ could affect N- to C-terminal interaction within BicD. BicD^PA66^ tracking results could suggest that there is an abrupt change from a closed to an open state sometime near phase IIb, followed by a clear inability down-regulate the open/active state once it has occurred, as seen in phase III (C).

Instead, we hypothesize that during normal wild-type development, BicD's motor regulatory functions can be auto-inhibited by an intramolecular BicD-BicD interaction, but that the PA66 point mutation has altered this interaction. Our model (Fig. 7) is as follows. First, we hypothesize that BicD can move between two main conformations, a ‘closed’ conformation (Fig. 7a, left) and an ‘open’ conformation (Fig 7a, right). Such a range of conformations is consistent with past EM studies visualizing BicD [10] (however, we do not attempt to differentiate between the possibility that intermediate states could reflect either the modulation of time spent in the completely-open vs completely-closed states, or the possibility that an intermediate conformation could be adopted for a given period of time). Secondly, it has been previously suggested [8], [12] that BicD may be capable of self-inactivation by positioning its N- and C-terminal ends near one another. In our case, we propose that when BicD is in its ‘closed’ state, it cannot regulate/activate either the plus-end or minus-end motors, but that it is maximally able to activate such motors when in an entirely ‘open’ state. This hypothesis builds on previous observations that fragments of BicD can interact with dynein [7], [8], [13] and kinesin [19], but that this ability can be absent or minimized in the full-length protein. The functional relevance of such conformational changes are consistent with the observation that while the N-terminus is generally considered the dynein recruiting fragment, there are also dynamitin (dynactin) and dynein intermediate chain (D.I.C.) interacting regions of BicD at the C-terminal portion of BicD (Fig 1B).

From our data studying the PA66 mutation, we further hypothesize that the BicD-BicD inhibitory interaction is itself actively regulated during development, and the PA66 mutation alters the regulation of the interaction (Fig 7C). Our model is that during wild type development, with normal BicD function, run-lengths are in part controlled by the amount of time BicD spends in the ‘open’ configuration–or alternatively, how ‘open’ it is (Fig. 7B). Thus, the extremely long runs in phase IIB would reflect BicD being completely ‘open’, and both phase II and phase III would reflect slightly more closed forms of BicD–or alternatively, BicD spending more time in a ‘closed’ configuration. Implicit in this model is the hypothesis that plus-end motion is more sensitive to exact BicD levels than minus-end motion: in phase II and III there is a basal level of BicD activity sufficient to achieve a fixed level of minus-end transport, but to really up-regulate minus-end transport requires the ‘completely open’ conformation found in IIB. In contrast, the moderately higher level of BicD activity in phase II relative to phase III (perhaps due to higher overall levels of the protein present on the droplet) is enough to increase plus-end motion. Such a sensitivity of plus-end motion to exact BicD levels/activity is consistent with the larger response of plus-end motion to the slightly increased BicD levels in t = 1 vs t = 3 embryos (see Fig. 3A, B).

Interpreting the phenotype of BicD^PA66^ within this general framework suggests that the point mutation affects the regulation of the closed versus open states of BicD, but requires that whatever role BicD has in the initial recruitment of dynein (if any) is not severely altered by the mutation (since there are more moving droplets in the BicD^PA66^ embryos than in the t = 1 or t = 3 embryos). We hypothesize that in contrast to normal Phase II BicD which spends some time partly open, phase II BicD^PA66^ is unable to respond to regulatory signals that keep it partly open. It would therefore be locked completely in the ‘closed’ state, and be unable to properly activate the motors. It thus acts as a ‘null’ mutant in phase II (i.e. the tracking phenotype is the same as for the t = 3 background). Then, for phase IIb, the mutant protein responds appropriately to a second regulatory event, that induces maximal opening of BicD in phase IIb. Consequently, the null phenotype disappears, and phase IIb motion is excellent; there is the suggestion that runs may be even longer than wild-type in the PA66 background. Finally, just as the mutant was unable to respond to whatever signal was present in phase II (to keep it partly open), in phase III it is unable to respond to the signal to partly close up, and remains fully open. Functionally, this causes BicD^PA66^ phase III run lengths to be quite similar to those of phase IIB, i.e. the expected significant decrease in phase III run lengths fails to occur. In conclusion, we believe that the BicD N-terminus/C-terminus interaction is regulated to control the amount that BicD increases plus-end and minus-end transport, and that this regulation is altered by the A40V mutation.

Our data shows that BicD function is essential for the ability to regulate bi-directional lipid droplet motion. BicD activity alters both plus-end and minus-end transport, and can play a role in balancing the relative contributions of each to allow regulated net transport. More than simply a physical attachment between dynein and the cargo, BicD plays interesting roles in regulating motion. It is interesting to speculate that BicD could do this by altering or facilitating the interaction of dynactin with dynein and possibly kinesin. Such a hypothesis is consistent with our past work and that of others implicating dynactin in coordinating opposite motors [26], [27], with the finding that for mRNA particles dynactin modulates dynein-mediated movement [25], and with the previous findings discussed above that BicD can bind both Dynactin and Dynein, thus in principle allowing it to act as an intermediary between the two. However, the extent that this occurs—and if so the mechanistic details of such a process—remains for future work to determine.

Analysis of the BicD^PA66^ point mutant demonstrates that BicD's dual abilities—to facilitate up-regulation of transport, and to facilitate switching the net direction of transport—are both regulated and separable. Clearly, a great deal of future work will be required to understand these functions at a molecular level, but the model proposed here should be helpful in guiding some of these new studies.

## Materials and Methods

### Fly Stocks and Crosses

Oregon-R was the wild-type stock. BicD^r6^/Df(2L)TW119; P[w^+^ hsp70 BicD]-94/+ served as our null for purposes of particle tracking. Our BicD^r6^ and Df(2L)TW119/CyO stocks were obtained from the laboratory of Beat Suter. The associated heat shock rescue protocol is based on that described in Swan and Suter 1996. Our slightly modified version involved one hour, once a day, 37° C heat shocks from early larval stages until adulthood. Heat shock rescued BicD^null^ females and their embryos were considered ‘t = 1’ one day after and ‘t = 3/4’ three/four days after cessation of the heat-shock regimen. The HA-tagged BicD transgene inserted on the first chromosome expresses approximately 12% of wild-type levels as previously described [11]. Therefore, a HA40::BicD/+; BicD^null^/Df female expressed approximately 6% of wild-type BicD levels. The HA40:BicD stock, H2Δ stock, and H3Δ stock were provided by the lab of Ruth Steward. Other stocks used in this study are publicly available on FlyBase (http://flybase.bio.indiana.edu). For the terminal cross resulting in embryos to be analyzed, Wild-type OrR males were used to fertilize the females, i.e. OrR males were crossed to (a) heat-shock rescue (null) females (i.e. BicD^r6^/Df119; Phs>BicD/+), to (b) “PA66 females” (HA40::BICD/+; PA66/PA66), and were also used for (c) all reclouding rescue experiments.

### Embryo Staging

Preliminary one hour embryo pre-collections were collected and discarded in order to eliminate fertilized eggs that females flies may have been storing. Timed phase specific purifications were based on one hour collection windows. Phase zero collections were stopped (e.g. lysed, fixed, etc.) immediately after collection. Phase I, II, and III collections were aged 1.5, 2.5, and 3.5 hours respectively following the one hour collection and then stopped. In contrast, embryos squashed for particle tracking were staged by eye. Phase II tracking was staged by identifying the point at which the cellularizing plasma membrane had moved halfway down the length of the syncytial nuclei. Phase IIb was defined as beginning when the membrane reached the basal edge of the nuclei. Phase III was defined as beginning at the initial dorsal movement of the pole cells which occurs early in embryogenesis.

### Isolation of Lipid Droplets by Subcellular Fractionation

Lipid droplets were purified as described previously (Cermelli et al., 2006). In brief, embryo lysates were loaded beneath a sucrose gradient solution and ultracentrifuged. Afterwards, the buoyant lipid droplets were collected from the top of the gradient. Isolated lipid droplets were then solublized in SDS-containing buffer for subsequent SDS-PAGE analysis. The step gradient western seen in figure 2f is based on a scaled down version of the above mentioned purification protocol.

### Particle Tracking and Statistical Analysis

Droplet motion was recorded as previously described [28]. Particle tracking was performed using automated analysis of each video frame. Runs were defined as ending only when a reversal in the direction of transport was observed. Pauses were included as part of a continuous run if a reversal was not identified.

We used custom video tracking software to analyze our data (details to be published elsewhere). Briefly, we determined the location of lipid droplets in each video frame via template matching [29] and then used these records of droplet positions (versus frame number) to construct motion tracks for each particle. Because lipid droplets are extended objects in our videos (∼500 nm in diameter), tracks could be unambiguously assembled based on particle proximity in subsequent frames. Tracks were then filtered to identify droplets which moved extended distances over many frames along a line perpendicular to the edge of the embryo [16].

Here, as a quantitative measure of overall motion of droplets, for each field of view (for all movies of a given genetic background) we also report the ratio of the droplets moving directionally (1 um minimum travel length) to the total number of detected droplets (no minimum travel length requirement). This latter quantity was determined as above, however no filtering was performed on the tracks.

### MT staining and Lipid Droplet Immunoflouresence

Microtubule staining of embryos involved dechorionating in 50% bleach, rinsing in water and fixing in a mix of heptane and 50/50 PBS/formaldehyde concentrate (37% formaldehyde) for one hour. The PBS/fixative phase was then removed and replaced with methanol and the solution was shaken to induce devitellinization of the embryos. These were stored overnight in methanol. Embryos were then rehydrated in PBT (PBS+0.2% Tween) and blocked in PBT+10% BSA. Mouse monoclonal anti-alpha-tubulin ‘AA4.3’ (DSHB) and goat-anti-mouse Alexa 488 were used to stain microtubules. Embryos were then stored and visualized in Vectashield mounting medium (Vector Laboratories).

Immunoflourescent labeling of lipid droplets was performed in whole fixed embryos. Embryos were dechorionated in 50% bleach, rinsed in water, and then fixed for 40 minutes in a 50/50 mix of heptane and 10% Formalin solution (3.7% formaldehyde). Embryos were filtered out of fixative with a nylon mesh and then hand de-vitellinized in a dish of PBS+1% BSA. BicD immunoflouresnce was accomplished with the mouse monoclonal anti-BicD antibody 4C2 (DSHB) at 1∶500 and a goat-anti-mouse Alexa 488 conjugated secondary antibody (Invitrogen) at 1∶1000. Lipid droplets were identified using either a 12 µg/mL Nile Red solution or by using the lipid droplet specific rabbit polyclonal LSD2-3b at 1∶500 and a goat-anti-rabbit Alexa 568 at 1∶1000 as the secondary. All blocking and incubations were done in PBS+10% BSA. Confocal imaging was done on a LSM 510 Meta laser scanning microscope (Zeiss).

### Sequencing of BicD^PA66^

Genomic DNA was obtained from sterile PA66 homozygote female flies by extraction, phenol/chloroform purifiction, and then ethanol precipitation. Two PCR products were generated to exclude the sequencing of a 7.5 kb first intron. The most 5′ fragment was 400 bp and the most 3′ fragment was approximately 3 kb. PCR products were then sent out for sequencing (Genewiz DNA sequencing service).
